# Transitioning from cerebrospinal fluid to blood tests to facilitate diagnosis and disease monitoring in Alzheimer's disease

**DOI:** 10.1111/joim.13332

**Published:** 2021-06-26

**Authors:** D. O. T. Alawode, A. J. Heslegrave, N. J. Ashton, T. K. Karikari, J. Simrén, L. Montoliu‐Gaya, J. Pannee, A. O´Connor, P. S. J. Weston, J. Lantero‐Rodriguez, A. Keshavan, A. Snellman, J. Gobom, R. W. Paterson, J. M. Schott, K. Blennow, N. C. Fox, H. Zetterberg

**Affiliations:** ^1^ From the Department of Neurodegenerative Disease UCL Queen Square Institute of Neurology London UK; ^2^ UK Dementia Research Institute at UCL London UK; ^3^ Department of Psychiatry and Neurochemistry Institute of Neuroscience and Physiology The Sahlgrenska Academy University of Gothenburg Gothenburg Sweden; ^4^ Wallenberg Centre for Molecular and Translational Medicine Department of Psychiatry and Neurochemistry Institute of Neuroscience and Physiology The Sahlgrenska Academy at the University of Gothenburg Gothenburg Sweden; ^5^ Department of Old Age Psychiatry Institute of Psychiatry, Psychology & Neuroscience King’s College London London UK; ^6^ NIHR Biomedical Research Centre for Mental Health & Biomedical Research Unit for Dementia at South London & Maudsley NHS Foundation London UK; ^7^ Clinical Neurochemistry Laboratory Sahlgrenska University Hospital Mölndal Sweden; ^8^ Dementia Research Centre Department of Neurodegenerative Disease UCL Queen Square Institute of Neurology London UK; ^9^ Turku PET Centre University of Turku Turku Finland

**Keywords:** Alzheimer's disease, Blood, Cerebrospinal fluid, Diagnosis, Disease monitoring, Fluid biomarkers

## Abstract

Alzheimer’s disease (AD) is increasingly prevalent worldwide, and disease‐modifying treatments may soon be at hand; hence, now, more than ever, there is a need to develop techniques that allow earlier and more secure diagnosis. Current biomarker‐based guidelines for AD diagnosis, which have replaced the historical symptom‐based guidelines, rely heavily on neuroimaging and cerebrospinal fluid (CSF) sampling. While these have greatly improved the diagnostic accuracy of AD pathophysiology, they are less practical for application in primary care, population‐based and epidemiological settings, or where resources are limited. In contrast, blood is a more accessible and cost‐effective source of biomarkers in AD. In this review paper, using the recently proposed amyloid, tau and neurodegeneration [AT(N)] criteria as a framework towards a biological definition of AD, we discuss recent advances in biofluid‐based biomarkers, with a particular emphasis on those with potential to be translated into blood‐based biomarkers. We provide an overview of the research conducted both in CSF and in blood to draw conclusions on biomarkers that show promise. Given the evidence collated in this review, plasma neurofilament light chain (N) and phosphorylated tau (p‐tau; T) show particular potential for translation into clinical practice. However, p‐tau requires more comparisons to be conducted between its various epitopes before conclusions can be made as to which one most robustly differentiates AD from non‐AD dementias. Plasma amyloid beta (A) would prove invaluable as an early screening modality, but it requires very precise tests and robust pre‐analytical protocols.

## Graphical abstract

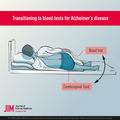

**Abbreviations**
AD: Alzheimer’s disease
Aβ: amyloid beta
APP: amyloid precursor protein
AT(N): amyloid, tau (neurodegeneration)
BBB: blood–brain barrier
CJD: Creutzfeldt–Jakob disease
CSF: cerebrospinal fluid
CU: cognitively unimpaired
DS: Down syndrome
ECL: electrochemiluminescence
ELISA: enzyme‐linked immunosorbent assay
FAD: familial Alzheimer’s disease
FDG‐PET: fluorodeoxyglucose positron emission tomography
MCI: mild cognitive impairment
MRI: magnetic resonance imaging
MS: mass spectrometry
NfL: neurofilament light chain
NFT: neurofibrillary tangle
NIA‐AA: National Institute of Aging and Alzheimer’s Association
PET: positron emission tomography
P‐tau: phosphorylated tau
SCD: subjective cognitive decline
Simoa: single molecule array
TBI: traumatic brain injury
T‐tau: total tau

## Introduction

### AD, biomarkers and the AT(N) criteria

**Fig. 1:**
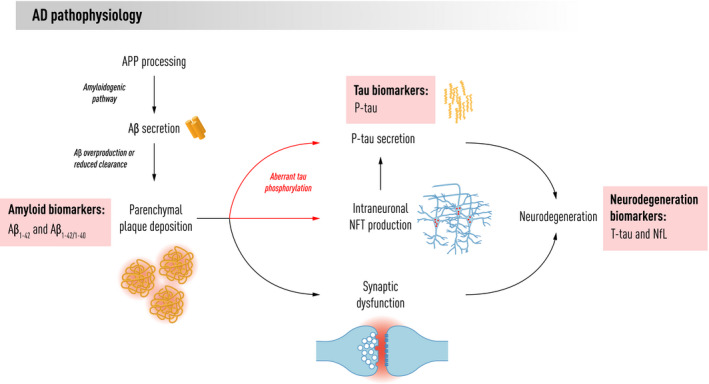
AD pathophysiology and AT(N) criteria fluid biomarkers.

Alzheimer’s disease (AD) is the most common form of dementia worldwide. It is characterized by (1) the presence of amyloid beta (Aβ) plaques in the brain parenchyma, which is often accompanied by Aβ in cerebral blood vessels (amyloid angiopathy); (2) intraneuronal neurofibrillary tangles (NFTs), composed of hyperphosphorylated tau; and (3) neurodegeneration [1, 2, 3]. According to the amyloid cascade hypothesis, accumulation of misfolded Aβ years before clinical symptom onset is the initial trigger of AD pathogenesis [4]. This accumulation of Aβ, as well as the production of toxic oligomeric species, results in aberrant tau phosphorylation and misfolding, ultimately inducing neuronal loss and plaque‐induced synaptic dysfunction [5]. This pathophysiological process is summarized in Fig. 1. Histopathological analysis of the brain at autopsy remains the gold standard for definitively diagnosing AD. However, molecular biomarkers have been developed to increase the accuracy of diagnosing AD clinically [6].

A biomarker is a naturally occurring, detectable indicator that can be measured to assess a physiological or pathological state [7, 8]. The importance of biomarkers is highlighted in the recent update of the National Institute of Aging and Alzheimer’s Association (NIA‐AA) research framework in 2018, in which a clinical diagnosis of AD is supported by biomarker evidence of a disease‐specific pathophysiological signature, rather than by clinical symptoms alone [9]. A key reason for this is the inaccuracy of a diagnosis based solely on symptoms, with one multi‐centre study observing the sensitivity and specificity of clinically probable AD to detect Braak stages V/VI to be 76.6% and 59.5%, respectively [10, 11]. There are marked phenotypic differences within AD, especially in younger patients, and the symptoms overlap with other neurodegenerative disorders, including vascular dementia, and mood disturbances such as depression [12]. A secure diagnosis is important to ensure patients receive the correct management (of AD, or of alternative conditions), and to provide prognostic information, advice and support. Furthermore, it is now clear that histopathological changes predate symptom onset by several years in both familial and sporadic forms of AD [13, 14, 15, 16]. While not currently clinically indicated, in the future it may become important to make a diagnosis of AD before symptom onset – if a disease‐modifying treatment is shown to be effective at this early stage.

Detection of AD pathology pre‐symptomatically is already important for research and for clinical trials that seek to show disease modification at this stage. Clinical trials aiming to halt, or significantly slow, AD progression have thus far proven ineffective. This is possibly due to the inclusion of symptomatic patients who have progressed too far along the disease process, and in whom significant irreversible neuronal loss has already occurred [17]. Conversely, it may be due to some participants having a false AD diagnosis. This is particularly true of the solanezumab trial, where some recruited participants were later found to be amyloid PET‐negative, hence were unlikely to have AD [18]. Furthermore, the lack of success in recent clinical trials may be due to too short trial duration and is further complicated by some participants displaying AD mixed with other disease pathologies, rather than being pure AD cases. Identifying individuals with AD pathology years prior to symptom onset will enable recruitment into clinical trials at a much earlier, and potentially more tractable, disease stage, and hence may prove more effective at identifying treatments to slow, or perhaps even halt, the disease process. Moreover, as participants in such trials would not be displaying cognitive symptoms, conventional cognitive/symptomatic endpoints are unlikely to be effective for identifying response to treatment, and so dynamic biomarkers which are sensitive to progression in pre‐symptomatic disease will be important. Table 1 summarizes the use of available CSF and neuroimaging biomarkers in clinical trials, along with upcoming blood‐based biomarkers.

**Table 1:** Biomarker use in AD clinical trials

Intended use in trial	CSF biomarkers	Neuroimaging biomarkers	Blood biomarkers
Pre‐screening			NfL, p‐tau, Aβ_1‐42_
Supporting diagnosis	T‐tau, p‐tau, Aβ_1‐42_	Amyloid PET, Tau PET	
Drug effect monitoring	*Dependent on the mechanism of action of the drug*	*Dependent on the mechanism of action of the drug*	*Dependent on the mechanism of action of the drug*
Safety markers	Markers of inflammation and BBB integrity	MRI	NfL, markers of inflammation

There are two main types of biomarkers for molecular AD brain changes – neuroimaging biomarkers (primarily positron emission tomography [PET] imaging) and fluid biomarkers (primarily cerebrospinal fluid [CSF]) [19]. The AT(N) criteria for AD diagnosis, which divide seven AD biomarkers into three groups based on the pathophysiological characteristic of AD they measure, include both of these classes of biomarkers [20] and are summarized in Table 2, where we also list a number of upcoming blood biomarkers. ‘A’ refers to Aβ pathology, as depicted by increased amyloid PET uptake, decreased CSF Aβ 1‐42 (Aβ_1‐42_) or decreased Aβ_1‐42_/Aβ_1‐40_ ratio (Aβ_1‐42/1‐40_). ‘T’ refers to tau pathology, as depicted by positive tau PET tracer uptake or increased CSF phosphorylated tau (p‐tau). Finally, ‘(N)’ refers to neurodegeneration or neuronal injury, as depicted by decreased signal on [^18^F]‐fluorodeoxyglucose (FDG)–PET, grey matter atrophy on structural magnetic resonance imaging (MRI), increased CSF total tau (t‐tau) or increased CSF neurofilament light‐chain (NfL) [20]. ‘(N)’ is denoted in brackets to highlight that the biomarkers of neuronal injury are not specific to AD [9]. The fluid biomarkers in the AT(N) criteria can be seen alongside the pathophysiological process they reflect in Fig. 1.

**Table 2:** Summary of AT(N) criteria biomarkers

Criteria aspect	Pathology	Neuroimaging biomarkers	CSF biomarkers	Blood biomarkers
A	Aβ	Amyloid PET	Aβ_1‐42_ or Aβ_1‐42/1‐40_	Aβ_1‐42/1‐40_
T	Tau	Tau PET	P‐tau	P‐tau
(N)	Neurodegeneration	MRI or FDG‐PET	T‐tau or NfL	NfL

While the AT(N) criteria highlight that both neuroimaging and fluid biomarkers can reliably confirm pathophysiological evidence of AD, fluid biomarkers offer the advantage of being able to detect the presence of multiple molecular pathologies in one bio‐sample, as well as being of lower cost. However, a drawback of fluid biomarkers is the lack of anatomical information on the location and extent of pathologies, which can be gained from neuroimaging. Indeed, fluid biomarkers reflect a pathological process in the tissue, while neuroimaging, with a few exceptions, quantifies this pathology [21]. In this review, using the AT(N) criteria as a framework, we will address the evidence behind current CSF‐based biomarkers for AD, with a particular focus on those that have potential for translation into blood‐based biomarkers.

## CSF and blood biomarkers for AD‐related pathologies

Before delving into potential blood‐based biomarkers for AD, it is important to consider some advantages and potential drawbacks common to all. Although CSF has the advantage of being in direct contact with the cerebral extracellular space, blood is less invasive to collect. Consequently, it is more suitable for obtaining repeated measurements from patients and is more easily accessible in low‐resource and non‐specialist settings worldwide [22, 23, 24]. While blood‐based biomarkers have the potential to function as an initial diagnostic screening tool in a primary care setting, prior to more in‐depth investigations in specialist centres [22, 25], measuring biomarkers of brain diseases in the blood is not without its challenges, namely (1) analyte concentrations are 10‐ to 100‐fold lower in the blood compared with CSF as a direct consequence of the blood–brain barrier (BBB) [26]; (2) some AD biomarkers are expressed by extra‐cerebral tissues; (3) proteases in the blood may break down analytes of interest prior to their measurement [27]. This puts extra demand on the pre‐analytical and analytical processes of relevance to blood biomarker measurements for CNS diseases.

### Amyloid beta

#### Aβ_1‐40_, Aβ_1‐42_ and Aβ_1‐42/1‐40_ as amyloid biomarkers in CSF

Aβ in CSF is already well established as a biomarker for AD. Aβ is produced when amyloid precursor protein (APP) is processed along its plaque‐forming (amyloidogenic) pathway. In this pathway, APP undergoes cleavage, first by β‐secretase followed by γ‐secretase, to produce an Aβ peptide [28]. The length of the Aβ peptide is dependent on the site (or extent) of γ‐secretase cleavage [29]. While Aβ peptides of varying amino acid lengths can be produced, the most abundant isoforms in CSF are Aβ_1‐38_, Aβ_1‐40_ and Aβ_1‐42_ [30], with Aβ_1‐40_ and Aβ_1‐42_ being the most widely studied isoforms. All Aβ peptides differ in amino acid sequence mainly at the C terminus [31].

Initial studies looking at total CSF Aβ in AD compared with controls had mixed results. While some showed a slight decrease in AD [32, 33, 34, 35], others found no change in total CSF Aβ concentration in AD compared with controls [36, 37, 38]. A major shift occurred following the discovery of Aβ_1‐40_ and Aβ_1‐42_ and the development of assays that are specific to these peptides. Investigations into the key differences between them revealed that Aβ_1‐42_ is more hydrophobic and hence is more prone to aggregation than Aβ_1‐40_ [31]. Furthermore, CSF concentrations of Aβ_1‐40_ remain unchanged in AD, whereas CSF concentrations of Aβ_1‐42_ decrease [39, 40, 41], suggesting that of the two, Aβ_1‐42_ provides a better biomarker for AD.

While CSF Aβ_1‐42_ concentrations have proven invaluable in diagnosing patients with probable AD dementia, Aβ_1‐42_ concentrations are to some extent dependent on the total Aβ concentrations of each patient [42]. Although it is necessary to have a threshold concentration of CSF Aβ_1‐42_ concentrations, below which an AD diagnosis is likely, inter‐individual differences make these thresholds somewhat arbitrary. Looking at CSF Aβ_1‐42_ concentrations alone may result in some patients being misdiagnosed as ‘normal’ when in fact concentrations may be abnormally low, if the CSF results had been related to their overall Aβ production and vice versa [43]. Harnessing the fact that CSF Aβ_1‐40_ concentration is not altered in AD, but instead may provide a useful index of an individual’s rate of Aβ production more generally, using CSF Aβ_1‐42/1‐40,_ may improve the reliability of results compared to using CSF Aβ_1‐42_ alone. Lewczuk *et al*. [40] found measuring CSF Aβ_1‐42/1‐40_ alongside Aβ_1‐42_ to improve diagnostic accuracy when comparing patients with AD to either controls or those with non‐AD dementias. Although the differences in diagnostic accuracy between Aβ_1‐42/1‐40_ and Aβ_1‐42_ were not statistically significant, likely due to low patient numbers. Additionally, Slaets *et al*. [41] reported that the addition of CSF Aβ_1‐42/1‐40_ to a biomarker panel for AD diagnosis consisting of Aβ_1‐42_, Aβ_1‐40_ and tau phosphorylated at threonine 181 (p‐tau_181_) significantly improved diagnostic accuracy compared with the same panel without Aβ_1‐40_ and Aβ_1‐42/1‐40_. However, it is worth noting that they observed no statistically significant difference in the area under the receiver operating characteristic curves between Aβ_1‐42_ and Aβ_1‐42/1‐40_. Furthermore, Struyfs *et al*. [30] and Bousiges *et al*. [44] both found that the addition of Aβ_1‐42/1‐40_ improved the ability to differentiate AD from non‐AD dementias, particularly frontotemporal lobe dementia and dementia with Lewy bodies. In non‐shunted normal pressure hydrocephalus, all Aβ peptides are reduced in CSF and measuring CSF Aβ_1‐42_ alone would result in a false positive, while the Aβ_1‐42/1‐40_ corrects for this [45]. Finally, the concordance of CSF Aβ_1‐42/1‐40_ with amyloid PET is higher than for CSF Aβ_1‐42_ alone [46], and the use of Aβ_1‐42/1‐40_ mitigates against adsorption effects that could lead to falsely low Aβ_1‐42_ [47, 48]. These studies clearly highlight the important role CSF Aβ_1‐42/1‐40_ plays in detecting Aβ pathology in AD.

#### Aβ_1‐40_, Aβ_1‐42_ and Aβ_1‐42/1‐40_ as amyloid biomarkers in blood

Building on the success of CSF Aβ_1‐42_ and Aβ_1‐42/1‐40_ in diagnosing AD, Aβ is an attractive blood‐based biomarker of AD because it easily crosses the BBB [49]. However, early investigations into the use of plasma Aβ_1‐42_ and Aβ_1‐42/1‐40_ as predictors of future AD development showed inconsistent results, with some reporting that high plasma Aβ_1‐42_ concentrations or a high Aβ_1‐42/1‐40_ are risk factors for AD development, while others reported the opposite, and still others reported no significant differences in plasma Aβ_1‐40_ and Aβ_1‐42_ between AD cases and controls [50, 51, 52, 53, 54]. The potential reasons for this include the following: the limited analytical sensitivity of the enzyme‐linked immunosorbent assay (ELISA)‐based techniques in use at the time; sub‐optimal or variable sample handling protocols; and, in many cases, the use of clinical criteria for diagnosis rather than evidence for Aβ pathology.

Recent advances in immunoassay technology to detect and quantify single protein measurements have increased their analytical sensitivity and have made it possible to quantify protein biomarkers at subfemtomolar concentration levels. There have been three main developments that have allowed for this. One has been to replace the enzyme label of the detection antibody with a molecule that emits light upon an electrochemical reaction, so‐called electrochemiluminescence (ECL) [55]. The second is a refinement of the basic ELISA technology, so‐called single molecule array (Simoa), compartmentalizing the detection reaction within femtolitre‐sized wells using magnetic beads onto which the immunocomplexes are captured, and digitalizing protein detection [56, 57, 58]. The final advancement has been the development of sensitive mass spectrometry (MS)‐based assays to quantify plasma Aβ peptides [59]. These technological advances have led to breakthroughs in efforts to detect and quantify Aβ present in peripheral blood.

A study by Janelidze *et al*. [25], which used ultrasensitive Simoa immunoassay technology to measure plasma Aβ_1‐40_ and Aβ_1‐42_ concentrations, found slight but significant correlations between plasma and CSF measurements of these analytes, but not of Aβ_1‐42/1‐40_. Furthermore, plasma Aβ_1‐40_, Aβ_1‐42_ and Aβ_1‐42/1‐40_ were all significantly decreased in AD patients compared with controls and patients with either mild cognitive impairment (MCI) or subjective cognitive decline (SCD). This was also observed in CSF, but the differences in CSF were much more pronounced. Additionally, plasma Aβ_1‐42/1‐40_ was lower in patients with MCI compared with both SCD and controls. The results from this study are in line with those seen in Rembach *et al*. [60], Jessen *et al*. [61] and Pesaresi *et al*. [62] and have been replicated by Vergallo *et al*. [63]. In addition to observing similar results to those above, Palmqvist *et al*. [64] showed that plasma Aβ_1‐40_, Aβ_1‐42_ and Aβ_1‐42/1‐40_ can accurately predict cerebral Aβ deposition. Of particular importance is a cross‐sectional study conducted by Palmqvist *et al*. [65], which highlights that plasma Aβ_1‐40_, Aβ_1‐42_ and Aβ_1‐42/1‐40_ reflect the changes seen in CSF, albeit not as dynamically, and that CSF and plasma Aβ alterations precede positive amyloid PET findings. While Chatterjee *et al*. [66] did not observe a significant difference in plasma Aβ_1‐40_ and Aβ_1‐42_ concentrations between the Aβ‐positive (Aβ^+^) and Aβ‐negative (Aβ^‐^) groups, perhaps due to their small sample size, they did observe a significantly lower plasma Aβ_1‐42/1‐40_ in the Aβ^+^ group compared to the Aβ^‐^. Finally, in a study which observed the utility of blood biomarkers without classification of CSF and PET, Simrén *et al*. [67] demonstrated significantly lower Aβ_1‐42/1‐40_ in AD patients compared with MCI and controls, however no change between MCI and controls. Interestingly, Aβ_1‐42/1‐40_ was associated with longitudinal change in grey matter volume, which is more strongly seen in cognitively unimpaired (CU) individuals than impaired patients.

Similar success in blood Aβ measurements has been observed using MS, which, due to detecting analyte ions (or gas‐phase‐produced fragments thereof) at their specific mass‐to‐charge ratio with high accuracy, has a greater analytical specificity and selectivity compared with immunoassays. An important difference compared with immunoassays is that while MS methods for plasma Aβ rely on antibodies for enrichment of the low abundance of Aβ peptides, quantification in MS is antibody‐independent, as the stable isotope‐labelled synthetic Aβ peptide analogues, that are used as internal standards, are co‐enriched with the endogenous peptides [68]. Furthermore, because samples analysed by MS are typically handled under denaturing conditions, in aqueous‐organic solvents, results are less influenced by matrix effects [69, 70, 71]. Of note, Ovod *et al*. [72] highlighted that the half‐life of Aβ in plasma is one third that of CSF Aβ. Additionally, they observed lower absolute concentrations of plasma Aβ_1‐42_ and Aβ_1‐42/1‐40_ in the blood of Aβ^+^ individuals, suggesting that plasma Aβ concentrations correlate positively with CSF Aβ. Furthermore, Nakamura *et al*. [59] showed that plasma Aβ_1‐42_ and Aβ_1‐42/1‐40_ accurately predicted amyloid PET positivity and negativity in two separate data sets, highlighting that plasma Aβ is inversely proportional to brain Aβ burden. Schindler *et al*. [73] observed similar results; however, they only saw a 10–15% change in plasma Aβ_1‐42/1‐40_ between amyloid PET‐positive and PET‐negative individuals, whereas in CSF, this change is 50%. Interestingly, direct (same‐sample) comparison of Simoa and MS‐based quantification of Aβ_1‐40_ and Aβ_1‐42_ in a preclinical cohort suggests that the correlation with brain amyloid pathology is higher with MS than with Simoa, at least at this stage of disease [74].

Despite this array of positive results, the contradictory results observed by other studies investigating plasma Aβ cannot be ignored. Consistent with early investigations into plasma Aβ, Giedraitis *et al*. [75] and Tamaoka *et al*. [76] reported no association between plasma Aβ_1‐40_ or Aβ_1‐42_ concentrations and AD pathology. Both Hansson *et al*. [77] and Lövheim *et al*. [78] agree with this finding, with Hansson *et al*. [77] also finding no correlation between plasma and CSF Aβ. One possible explanation for the contradictory results is the inter‐study variation in pre‐analytical practices [66], which has been addressed by the development of a standardized guideline for pre‐analytical variables in AD blood‐based biomarker research in 2015 [79]. Importantly, discrepancies between blood and CSF biomarkers may reflect sampling issues in both. A systematic review conducted by Hansson *et al*. [80] in 2018, looking at the variation in pre‐analytical methods for handling CSF samples prior to AD biomarker measurement, revealed a broad range of protocols was used in the 49 studies investigated. Out of the 15 variables assessed, the only two variables that remained consistent were the storage conditions (−80°C) and the lumbar puncture sampling location (L3‐5). In some cases, these variations have a significant effect on the biomarkers of interest and hence on results obtained from the study. For example, CSF Aβ_1‐42_ is significantly affected by storage tube type [81, 82, 83], and some studies have found that centrifuging CSF samples prior to analysis may cause significant reductions in CSF Aβ_1‐42_, likely due to the high propensity of Aβ_1‐42_ to aggregate [84, 85]. While Hansson *et al*. [80] focussed their review towards CSF samples and have recently published an updated standardized pre‐analytical protocol for measuring AD biomarkers in CSF [86], the results obtained in their 2018 review highlight the need for universal pre‐analytical protocols, not only for CSF, but also for blood sample handling.

Alternatively, these contradictory results may be due to the variation in patient cohort characteristics between studies. Aβ concentrations vary depending on the patient’s stage of disease, which reflects the increasing plaque burden as the disease progresses. This, combined with the fact that Aβ is ubiquitously expressed in extra‐cerebral tissues, may explain the variations in results obtained when investigating plasma Aβ concentrations. Indeed, a large proportion of plasma Aβ is not brain‐derived, resulting in a much lower (10‐15%) reduction in plasma Aβ_1‐42/1‐40_ compared with CSF Aβ_1‐42/1‐40_ (50%) [73]. A final possible explanation for the variation in results may be matrix effects caused by plasma proteins in the blood [87]. These matrix effects can be limited by the dilution of the plasma sample prior to analysis [25]. In fact, several studies have now shown that Aβ_1‐42/1‐40_ reflects cerebral Aβ pathology, provided it is determined using methods which minimize matrix effects, such as MS [59, 72, 88]. However, variations in analytical protocols and instruments used mean that this is not always done, resulting in greater interference caused by other molecules present in the plasma, and hence greater variation in results obtained across different studies.

Finally, some of the improvements in diagnostic performance recorded for plasma Aβ tests during recent years may be due to improved diagnostic work‐up of the study participants so that most of them have been classified as Aβ^+^ or Aβ^‐^ based on CSF or PET biomarkers. This has made it less likely that the control group contains individuals with preclinical amyloid pathology and that the AD group contains individuals with cognitive deterioration, having already ruled out non‐AD neurodegenerative diseases. Studies in memory clinic or population‐based cohorts without prior stratification by CSF or PET biomarkers should ascertain the true diagnostic potential of plasma Aβ, giving insight into its real‐world use.

#### Aβ_1‐43_ as a potential amyloid biomarker

While Aβ_1‐40_ and Aβ_1‐42_ remain the two most widely studied isoforms of Aβ, longer Aβ peptides, including Aβ_1‐43_, have been observed within the brains of AD patients. Early investigations into Aβ revealed that although Aβ_1‐42_ is the most abundant Aβ peptide in plaques, Aβ_1‐43_ comprises a minor component, with Aβ_1‐40_ predominantly being present in cerebral microvessels rather than in parenchymal plaques [89, 90]. However, recent studies have shown that Aβ_1‐43_ may play a greater role in AD than previously thought. Parvathy *et al*. [91] found that both Aβ_1‐42_ and Aβ_1‐43_ are associated with early disease progression, with deposition of both peptides being observed prior to AD diagnosis. Additionally, in mouse models of familial AD (FAD), Saito *et al*. [92] showed that not only does Aβ_1‐43_ have a greater propensity to aggregate and is more neurotoxic than Aβ_1‐42_, but it also accumulates in AD brains more frequently than Aβ_1‐40_, observations which are supported by the findings of Welander *et al*. [93] and Keller *et al*. [94]. Furthermore, Jäkel *et al*. [95] observed a positive correlation between Aβ peptide length and plaque load (Aβ_1‐43_ > Aβ_1‐42_ > Aβ_1‐40_). These results deviate somewhat from the observations of Iizuka *et al*. [89], who found Aβ_1‐42_ to be the major component of plaques, with Aβ_1‐43_ being a minor component, and Aβ_1‐40_ only being present in cerebrovascular amyloid. These differences in results are possibly due to the very small cohort size used by Iizuka and colleagues. Similarly, Perrone *et al*. [29] found CSF Aβ_1‐43_ to have a positive correlation with Aβ_1‐42_ concentrations, with CSF Aβ_1‐43_ concentrations being significantly reduced in FAD mutation carriers. These studies highlight that Aβ_1‐43_ plays a role in AD, albeit less well investigated.

Despite the above evidence, there remains very little published literature on attempts to produce a functioning biomarker assay for Aβ_1‐43_ in AD. One reason for this is that Aβ_1‐43_ has a very similar diagnostic accuracy to CSF Aβ_1‐42_; hence, it is unlikely to provide additional diagnostic value over existing biomarkers [96, 97]. However, Aβ_1‐43_ may prove useful in differentiating between different groups of AD patients. One study observed a significantly greater reduction in CSF Aβ_1‐43_, but not Aβ_1‐42_, in early‐onset AD compared with late‐onset AD [97], while another study showed that Aβ_1‐43_, but not Aβ_1‐42_, could identify amnestic MCI patients who progressed to AD [98]. In addition, Lauridsen *et al*. [98] observed a significant decrease in CSF Aβ_1‐43_ over the 2‐year follow‐up period, with no significant difference seen in CSF Aβ_1‐42_ concentrations. It is clear that Aβ_1‐43_ plays a role in AD; hence, there is a need to investigate this peptide further, particularly in blood.

### Phosphorylated tau

Tau is a microtubule‐associated protein that is a natural component of healthy, mature neurones [99]. A very small percentage of tau may be phosphorylated in healthy individuals. However, in AD, tau is 3‐4 times more phosphorylated and aggregates intraneuronally into NFTs composed predominantly of p‐tau [99, 100, 101]. Tau was first identified as a CSF biomarker for AD in 1993 using ELISA [102]. Since 1993, ELISA methods for measuring t‐tau that detect all tau isoforms, irrespective of their phosphorylation, have been developed. Along with the 6 different isoforms of tau in the CNS, produced by alternate splicing, there are up to 85 possible tau phosphorylation sites [103]. Studies have revealed that the concentration of p‐tau in CSF accurately depicts the extent of p‐tau deposition within the AD brain [104], and in contrast to t‐tau, there is essentially no change in concentrations of certain p‐tau species in other neurological conditions like acute stroke [105] or Creutzfeldt–Jakob disease (CJD) [106], nor in other tauopathies and neurodegenerative diseases [107, 108, 109, 110, 111]. This suggests that several p‐tau species are specific to AD when measured in biofluids, and can be used to distinguish AD from other neurodegenerative disorders. It is thought that both p‐tau and t‐tau increase in CSF as a direct response to Aβ pathology, as opposed to being markers of neuronal loss, as previously assumed [88, 112]. Rather, it may be the resultant tau pathology caused by Aβ‐induced tau secretion that causes neurodegeneration in AD, since neurodegeneration and cognitive loss do not occur in the absence of tau [113]. This is consistent with earlier studies in mouse models, which show increases in CSF endogenous murine tau concentration without evidence of neuronal loss in APP transgenic mice [114]. In addition to phosphorylation, increasing evidence indicates that both N‐glycosylation and O‐glycosylation are implicated in AD, emphasized by the fact that tau carries potential N‐glycosylation and O‐glycosylation sites [115]. However, no established biomarkers to study the pathophysiological relevance of this in humans exist yet. In this section, we will discuss tau phosphorylated at three sites – threonine 181 (p‐tau_181_), threonine 217 (p‐tau_217_) and threonine 231 (p‐tau_231_).

#### P‐tau181, 217 and 231 as tau biomarkers in CSF

Early studies looking at CSF p‐tau concentrations in AD using ELISA revealed that irrespective of which p‐tau epitope was measured, p‐tau is significantly elevated in AD compared with age‐matched CU controls, as well as patients with non‐AD dementias [109, 110, 116, 117, 118]. Further investigations into the efficacy of combining p‐tau measurements with CSF Aβ_1‐42_ and/or Aβ_1‐42/1‐40_, and CSF t‐tau have led to CSF p‐tau, particularly p‐tau_181_, being included in the AT(N) criteria for AD diagnosis and the NIA‐AA research framework for defining AD [9, 20]. However, more recently, there has been question as to whether certain p‐tau epitopes function better than others as AD biomarkers.

Of all the p‐tau epitopes, immunoassays detecting CSF p‐tau_181_ are by far the most widely studied. Unless otherwise specified, ‘p‐tau’ is almost always assumed to refer to mid‐region p‐tau_181_ [119, 120]. However, CSF is known to predominantly contain a mixture of both N‐terminal and mid‐region tau fragments, with C‐terminal fragments being relatively scarce [121, 122, 123]. CSF p‐tau_181_ has proven useful in differentiating AD from controls and other tauopathies and neurodegenerative diseases, while also predicting cognitive decline in preclinical cases of AD [124, 125, 126]. However, in 2020, two separate studies – one using ELISA [127] and the other using MS [128] – observed that CSF p‐tau_217_ displayed a larger‐fold change with AD pathology than p‐tau_181_. A third study concluded that CSF p‐tau_217_ serves as a better marker of cognitive decline than CSF p‐tau_181_ [129], and a fourth study, using a novel ultrasensitive immunoassay on the Simoa platform, observed much less overlap between diagnostic groups (AD vs controls and amyloid PET‐positive vs amyloid PET‐negative) with p‐tau_217_ than with p‐tau_181_ [130]. In summary, these studies argue that p‐tau_217_ is the superior tau pathology biomarker; therefore, it should be used more widely in clinical practice. Both Janelidze *et al*. [127] and Barthelemy *et al*. [128] observed that while CSF p‐tau_181_ clearly distinguished AD from the non‐AD groups studied, CSF p‐tau_217_ more markedly distinguished between the groups, and it showed a stronger correlation with tau PET and amyloid PET in AD patients.

To investigate these results further, Karikari *et al*. [131] conducted a head‐to‐head comparison of novel CSF p‐tau_217_ and p‐tau_181_ biomarkers, containing the N‐terminal amino acid 6‐18 epitope (N‐p‐tau_217_ and N‐p‐tau_181_, respectively), with the performance of already established p‐tau_181_ biomarkers, which target the mid‐region epitopes (mid‐p‐tau_181_), in AD and MCI patients in three cohorts. In their two validation cohorts, N‐p‐tau_217_ and N‐p‐tau_181_ increased in MCI‐AD patients, whereas mid‐p‐tau_181_ remained within normal range. Additionally, N‐p‐tau_217_ and N‐p‐tau_181_ both equally identified increased Aβ pathology and differentiated MCI‐AD from non‐AD MCI and Aβ^‐^ CU individuals significantly better than mid‐p‐tau_181_. The performance of N‐p‐tau_217_ and N‐p‐tau_181_ was virtually indistinguishable from one another, suggesting that CSF p‐tau_217_ may not be a more accurate biomarker for AD pathology, but rather it functions better than the p‐tau_181_ biomarkers to which it was compared to – mid‐p‐tau_181_. Furthermore, N‐p‐tau_217_ and N‐p‐tau_181_ both increase in synchrony with Aβ pathology changes, whereas mid‐p‐tau_181_ increases at a later disease stage [120, 131, 132]. Interestingly, Emeršič *et al*. [133] found CSF p‐tau_217_ to also be elevated in both AD and CJD, suggesting that p‐tau_181_ is more specific to AD, and may serve to better confirm AD diagnosis.

Studies looking at CSF p‐tau_231_ have shown huge promise, with early investigations finding CSF p‐tau_231_ to identify AD with 85% sensitivity and 97% specificity [118], and more recent studies observing a more prominent increase in CSF mid‐p‐tau_231_ in AD compared with a gold standard mid‐p‐tau_181_ immunoassay [120]. Of particular importance is a study conducted by Ashton *et al*. [134], which observed that compared with CSF p‐tau_181_ and p‐tau_217_, CSF p‐tau_231_ was more sensitive to the earliest changes in parenchymal Aβ pathology before amyloid PET positivity had occurred.

#### P‐tau181, 217 and 231 as tau biomarkers in blood

The challenges of measuring biomarkers of brain diseases in the blood have already been mentioned above. Previously, the low concentrations of tau in blood made it difficult to measure. However, the development of ultrasensitive immunoassay technologies has mitigated these difficulties [17]. Nonetheless, there remains one specific challenge which appears to be particularly problematic for tau. Tau is extremely stable in CSF, whereas in blood, it has a very short half‐life (~10h) [88]. This could be due to proteases causing an increased rate of tau degradation [27, 88]. Indeed, several studies investigating plasma tau clearance following hypoxic brain injury have highlighted the efficient clearance mechanisms of tau in blood [135, 136]. However, it is possible to minimize tau degradation by adopting fast and efficient pre‐analytical sample processing measures.

In one of the first studies of its kind, Shekhar *et al*. [137] attempted to quantify serum p‐tau_181_ in a small pilot study, consisting of AD dementia, MCI and control groups. They observed an elevated concentration of p‐tau_181_ in both the AD and MCI groups compared to controls, as well as in AD compared to MCI. Shortly after, in another pilot study, Tatebe *et al*. [138] attempted to quantify plasma p‐tau_181_ in AD dementia, Down syndrome (DS) and control groups, using a novel p‐tau_181_ Simoa assay which detects N‐p‐tau_181_. They observed a significantly higher concentration of p‐tau_181_ in both the AD and DS groups compared to their respective age‐matched controls, as well as a strong correlation between plasma and CSF p‐tau_181_ concentrations. These findings have been further corroborated by other studies in CU individuals and those with AD dementia, MCI and non‐AD dementias [139, 140, 141, 142, 143]. In a much larger‐scale study, Mielke *et al*. [139] found that plasma p‐tau_181_ was more strongly associated with Aβ and tau PET imaging than plasma t‐tau, and more sensitively and specifically predicted increased brain Aβ concentrations. This was further corroborated in a recent multi‐centre study conducted by Karikari *et al*. [143], which showed that not only can p‐tau_181_ identify AD with high diagnostic accuracy, but it also increases minimally in individuals diagnosed with AD but who are amyloid PET‐negative, and increases more prominently in individuals with decreased CSF Aβ prior to amyloid PET positivity. Moreover, Janelidze *et al*. [140] showed that plasma p‐tau_181_ can accurately predict future progression to AD dementia in individuals who were initially CU. In a longitudinal study, Lantero‐Rodriguez *et al*. [144] observed that plasma p‐tau_181_ accurately predicts AD pathology and discriminates between AD and non‐AD pathology, at least 8 years prior to death and subsequent neuropathological diagnosis. Similarly, O’Connor *et al*. [145] observed, in their longitudinal study of FAD, that plasma p‐tau_181_ concentrations were higher in mutation carriers than non‐carriers from 16 years prior to estimated symptom onset. Furthermore, Moscoso *et al*. [146] have recently shown that longitudinal changes in plasma p‐tau_181_ are associated with longitudinal neurodegeneration in AD‐specific brain regions, as measured by FDG‐PET and grey matter volume. Together, this evidence suggests plasma p‐tau_181_ poses a promising blood‐based biomarker for both AD diagnosis and for patient recruitment into clinical trials. Furthermore, it may provide longitudinal information relating to AD‐specific neurodegeneration that could be employed as a treatment response measure in therapeutic clinical trials.

Studies into the utility of plasma p‐tau_217_ in AD diagnosis began relatively recently but have had promising results. An investigation into core CSF and blood AD biomarkers in relation to amyloid PET revealed that plasma and CSF p‐tau_217_ concentrations change simultaneously [65]. Following on from this, one cohort study found plasma p‐tau_217_ to be increased in CU individuals with abnormal (i.e. positive) amyloid PET but normal tau PET, suggesting changes in plasma p‐tau_217_ precede the detectability of insoluble tau aggregates by tau PET [147]. Before conclusions can be made as to whether plasma p‐tau_217_ will function as a useful biomarker for early AD pathology, investigations must first be conducted to compare plasma p‐tau_217_ in AD with other neurodegenerative diseases, particularly CJD, since CSF p‐tau_217_ was found to be increased in this condition [133].

A recent study also demonstrates the high diagnostic performance of p‐tau_231_ in blood [148]. While at the cognitive impairment stage p‐tau_181_ and p‐tau_231_ are seemingly similar in diagnostic accuracy, the p‐tau_231_ epitope begins to increase early in the preclinical stage of the disease, similar to the findings in CSF [148]. The early increase is suggested to be a response to accumulating amyloid pathology under a threshold of amyloid PET positivity.

### Neurodegeneration

#### T‐tau as a neurodegeneration biomarker in CSF

CSF t‐tau in AD has been proposed to reflect the severity of Aβ‐induced neurodegeneration and neuronal or axonal injury [49, 140]. As with p‐tau, high concentrations of t‐tau have been observed consistently in AD patients [119]. Changes in CSF t‐tau are not specific to AD, as t‐tau is also increased in other cases of neuronal injury, including stroke, traumatic brain injury (TBI) and CJD [49]. However, recent studies have suggested that the t‐tau being measured in AD biofluids is secreted alongside p‐tau, and reflects Aβ‐induced tau secretion from living neurones [112]. While these neurones will eventually degenerate and die, the t‐tau being measured in AD is not thought to be a direct marker of this [149]. In contrast, the high CSF t‐tau with normal CSF p‐tau, measured in conditions like stroke, TBI and CJD, is a direct result of massive neuronal death, and in these cases, t‐tau is a marker of neuronal injury [149]. Therefore, in combination with raised p‐tau, increased CSF t‐tau does reflect AD pathology, rather than simply being a non‐specific effect of neuronal damage.

#### T‐tau as a neurodegeneration biomarker in blood

One of the earliest studies investigating plasma t‐tau in AD yielded discouraging results, reporting no significant increase in plasma t‐tau being seen in AD compared to non‐AD dementias [150]. However, this study was most likely limited by the low sensitivity of the ELISA technology used. Since the development of more sensitive ELISA technology, particularly through the use of Simoa, numerous studies have reported increased plasma t‐tau concentrations in AD [17, 136, 151, 152], with some observing a strong correlation between plasma and CSF t‐tau [151], and others observing a weak [152] or absent correlation [136]. Furthermore, one study reported reduced plasma t‐tau concentrations in AD [153]. While the general consensus is that plasma t‐tau concentrations increase in AD, Zetterberg *et al*. [136], Dage *et al*. [17] and Mattsson *et al*. [152] all observed significant overlap in plasma t‐tau ranges between their AD and non‐AD groups, including age‐matched CU controls. An additional study found an association between elevated plasma t‐tau concentrations and cognitive decline; however, this was independent of elevated brain Aβ [154]. It is possible that the inconsistent results thus far in measuring plasma t‐tau may be due to the currently available assays measuring a form of tau that is particularly susceptible to protease degradation [140]. Interestingly, Pase *et al*. [155] showed in a multi‐centre study that plasma t‐tau can act as a risk‐stratifier for progression to AD dementia. One strength of this study was *post‐mortem* correlation with tau pathology observed in a subset of the cohorts investigated. Nonetheless, the current evidence suggests plasma t‐tau may not be a useful diagnostic blood biomarker for AD, but high concentrations may provide prognostic evidence of incident neurodegeneration, similar to the performance of a t‐tau assay using N‐terminal anti‐tau antibodies which were recently described [156, 157].

#### NfL as a neurodegeneration biomarker in CSF

Neurofilaments are an important structural component of the neuronal cytoskeleton [158], and one specific subunit of neurofilaments, NfL, is primarily expressed in large‐calibre myelinated axons [159]. Increased CSF NfL concentrations have been associated with white matter lesions and subcortical brain damage in AD [160], as well as other neurodegenerative and non‐neurodegenerative diseases [161]. Hence, NfL is not specific to AD, but it functions as an excellent biomarker for neuronal death and axonal loss. Furthermore, CSF NfL concentrations are significantly increased in AD compared to CU controls, serving as an accurate marker of progression from MCI to AD and reflecting neurodegeneration independent of Aβ pathology [119, 161, 162, 163, 164].

#### NfL as a neurodegeneration biomarker in blood

Interest in NfL as a blood biomarker came about in relation to longitudinal studies, due to blood being easier to sample serially than CSF. Following the development and validation of the first assay to reliably measure serum NfL concentrations in 2013 using ECL [165], more sensitive assays have been developed using Simoa technology [166]. Indeed, in a comparison between three analytical platforms – ECL, standard ELISA and Simoa – Simoa was found to be the most sensitive at quantifying serum NfL concentrations [167]. Using this ultrasensitive Simoa assay, Mattsson *et al*. [166] showed for the first time that plasma NfL correlates with CSF NfL, but also with other hallmarks of AD. Furthermore, blood NfL has high diagnostic accuracy for AD, and it is increased prior to symptom onset, making it a promising biomarker for neuronal injury in this disease. These results have since been corroborated by the vast majority of studies across both sporadic and familial disease [168, 169, 170, 171, 172, 173], with Schultz *et al*. [172] observing that similar to CSF NfL, plasma NfL concentrations correlate with white matter damage in the brain, and Ashton *et al*. [173] demonstrating that plasma NfL correlates strongly with the severity of NFT pathology in AD seen in *post‐mortem* analysis. Due to the lack of specificity of NfL for AD, its value is unlikely to be in differentiating AD from other neurodegenerative diseases, but rather to distinguish neurodegeneration (including AD) from non‐degenerative causes of cognitive impairment (e.g. primary psychiatric causes) [174, 175]. Additionally, it can be used as a non‐invasive screening tool to identify patients at risk of cognitive decline, as well as a dynamic biomarker to monitor treatment efficacy and to track disease progression.

#### T‐tau vs. NfL as neurodegeneration biomarkers in AD

Both t‐tau and NfL are useful markers of neurodegeneration in AD. CSF t‐tau has the added advantage of correlating with Aβ pathology changes [88, 112], which is not the case for CSF NfL [176]. However, the evidence presented suggests that NfL translates better into a blood biomarker for AD neurodegeneration than t‐tau. Indeed, plasma NfL is robust to even a 48‐h delay in centrifugation of whole blood, in contrast to the known issues with plasma tau being susceptible to degradation by proteases [177]. Therefore, it is possible that plasma NfL may replace t‐tau in an initial blood‐based diagnostic work‐up for AD to confirm the presence of neurodegeneration, followed by CSF t‐tau being used in tertiary centres to aid the confirmation of Aβ‐induced neurodegeneration.

## An integrated hypothesis for AD pathogenesis

AD is an extremely complex disease. To date, research has shown that microglia are the primary mediators of neuroinflammation in AD brains. However, the role of neuroinflammation in AD pathogenesis remains highly debated. Some papers argue that neuroinflammation is neuroprotective, designed to clear Aβ plaques, while others argue that it is neurotoxic by promoting AD progression through cytokine release, phagocytosis of synapses and consequent neurodegeneration [178, 179, 180, 181, 182]. Furthermore, one review argues that microglia play both a neuroprotective and a neurodegenerative role, depending on the stage of AD [183].

In their recent review, Edwards [113] proposed a unifying hypothesis for AD pathogenesis, whereby they suggest the primary driver for AD progression following amyloid plaque deposition and Aβ‐induced synaptic damage is an inadequate microglial response. The authors introduce the idea that the magnitude with which microglia respond increases with disease progression, proposing that microglia are responsible for removing damaged synapses and hence play a neuroprotective role in AD. Consequently, this protective role of microglia prevents damage from propagating down the axon, thus breaking the cycle of Aβ‐induced synaptic dystrophy. This provides an alternative explanation for why some elderly individuals without dementia are found to have a similar burden of plaques and tangles to that seen in patients with clinically advanced AD at *post‐mortem* [184]. In essence, the plaque load an individual can tolerate prior to neurodegeneration occurring may be dependent on the genetic characteristics of their microglia, which determines the rate at which damaged synapses are phagocytosed [113].

A number of pathological mechanisms are addressed by Edwards [113], each of which present proteins which could function as fluid biomarkers for AD. In addition to Aβ, tau and NfL, these mechanisms and corresponding biomarkers include (1) markers of low‐level Aβ release (glutamate); (2) markers of dystrophic synapses (neurogranin, SNAP‐25, synaptotagmin); (3) markers of microglial activation (TREM‐2, YKL‐40); and (4) complement‐mediated synapse loss (complement proteins, *e.g*. C3). Tests for some of these proteins have shown promising results in CSF studies [185, 186, 187, 188], but translating them into blood tests will be difficult. Investigations have revealed that neurogranin [189] and soluble TREM‐2 [190] do not function well as blood biomarkers for AD. Additionally, YKL‐40 was found to be significantly increased in the AD and MCI groups compared to controls [191]. However, there was a significant overlap between the groups, and it did not correlate with CSF Aβ_1‐42_ or CSF p‐tau_181_. The proteins discussed by Edwards [113] are highly expressed in extra‐cerebral tissues. Consequently, any brain‐derived signal in blood is likely to be overwhelmed by release of proteins from other tissues.

## Conclusion

In conclusion, we have considered biomarkers which have the potential to be translated into blood biomarkers for AD. In particular, plasma p‐tau_181_ and NfL show huge promise, with both having significant evidence highlighting that assays for these markers work in both research laboratories and in specialist settings. Plasma NfL could potentially screen for a range of pathologies, not just AD, and act as a therapy response marker. As plasma p‐tau_181_ reflects both amyloid and tau pathology, it would be applicable in differential diagnoses compared to other dementias, as well as potentially functioning as a therapy response marker, given the changes seen in longitudinal studies. However, prior to clinical implementation, plasma p‐tau_181_ requires further analysis comparing assays targeting N‐terminal and mid‐region p‐tau_181_.

Plasma Aβ would have value in early, or even pre‐symptomatic, screening and recruitment to clinical trials. However, it would need cautious interpretation due to the prevalence of amyloid positivity increasing with age in individuals who will not develop AD in their lifetime. Nonetheless, the inter‐laboratory variation in pre‐analytical protocols has led to inconsistent plasma Aβ results. Therefore, a new standardized guideline for pre‐analytical variables in AD blood‐based biomarker research must be established for worldwide use, with implications for protocols which deviate from the proposed guideline.

Finally, plasma p‐tau_217_ and p‐tau_231_ studies look promising. However, more head‐to‐head comparisons of assays measuring different phospho‐forms of tau, using identical methods, are needed to reach a conclusion on which of these biomarkers most robustly separate AD from non‐AD neurodegenerative dementias.

Given the rapidly changing field, it is unclear which of these biomarkers will ultimately prove most useful to answer different clinical and research questions. As is often the case with technical advances, there are associated ethical issues, including the fact that the ease of testing with blood‐based measures may lead to inappropriate use, such as direct‐to‐consumer predictive testing without counselling or support being available. However, what is clear is that blood‐based biomarkers are set to transform both clinical and research practice – and will have wide, even global, applicability.

## Conflict of interest

DOTA has no conflicts of interest. JMS has received research funding and PET tracer from AVID Radiopharmaceuticals (a wholly owned subsidiary of Eli Lilly); has served as a consultant at advisory boards, or at data monitoring committees consulted for Roche, Eli Lilly, Biogen, Merck, GE and Axon Neuroscience SE; and is Chief Medical Officer for Alzheimer’s Research UK. KB has served as a consultant at advisory boards, or at data monitoring committees for Abcam, Axon, Biogen, JOMDD/Shimadzu, Julius Clinical, Lilly, MagQu, Novartis, Roche Diagnostics and Siemens Healthineers and is a co‐founder of Brain Biomarker Solutions in Gothenburg AB (BBS), which is a part of the GU Ventures Incubator Program. NCF has served as a consultant at advisory boards, or at a data monitoring committee for Roche, Biogen and Ionis. HZ has served at scientific advisory boards for Eisai, Denali, Roche Diagnostics, Wave, Samumed, Siemens Healthineers, Pinteon Therapeutics, Nervgen, AZTherapies and CogRx; has given lectures in symposia sponsored by Cellectricon, Fujirebio, Alzecure and Biogen; and is a co‐founder of Brain Biomarker Solutions in Gothenburg AB (BBS), which is a part of the GU Ventures Incubator Program.

## Funding

DOTA is supported by the International Journal of Experimental Pathology and the UK Dementia Research Institute at UCL. AOC acknowledges support from an Alzheimer’s Society Clinical Research Training Fellowship (AS‐CTF‐18‐001) and previous support from the Rosetrees Trust. JMS acknowledges the support of the National Institute for Health Research, University College London Hospitals Biomedical Research Centre, the Medical Research Council and the Alzheimer’s Association. KB is supported by the Swedish Research Council (#2017‐00915), the Alzheimer’s Drug Discovery Foundation (ADDF), USA (#RDAPB‐201809‐2016615), the Swedish Alzheimer Foundation (#AF‐742881), Hjärnfonden, Sweden (#FO2017‐0243), the Swedish state under the agreement between the Swedish government and the County Councils, the ALF agreement (#ALFGBG‐715986), the European Union Joint Programme for Neurodegenerative Disorders (JPND2019‐466‐236) and the National Institute of Health (NIH), USA (#1R01AG068398‐01). HZ is a Wallenberg Scholar supported by grants from the Swedish Research Council (#2018‐02532), the European Research Council (#681712), Swedish State Support for Clinical Research (#ALFGBG‐720931), the Alzheimer Drug Discovery Foundation (ADDF), USA (#201809‐2016862), the AD Strategic Fund and the Alzheimer's Association (#ADSF‐21‐831376‐C, #ADSF‐21‐831381‐C and #ADSF‐21‐831377‐C), the Olav Thon Foundation, the Erling‐Persson Family Foundation, Stiftelsen för Gamla Tjänarinnor, Hjärnfonden, Sweden (#FO2019‐0228), the European Union’s Horizon 2020 research and innovation programme under the Marie Skłodowska‐Curie grant agreement no 860197 (MIRIADE) and the UK Dementia Research Institute at UCL.
